# Current News

**Published:** 1892-08

**Authors:** 


### Current News.

### DENTAL LAW FOR THE DISTRICT OF COLUMBIA.

AN ACT FOR THE REGULATION OF THE PRACTICE OF DENTISTRY IN THE DISTRICT OF COLUMBIA, AND FOR THE PROTECTION OF THE PEOPLE FROM EMPIRICISM IN RELATION THERETO.

*Be it enacted by the Senate and House of Representatives of the United States of America in Congress assembled*, That it shall be unlawful for any person to practise dentistry in the District of Columbia unless such person shall register with the health officer in compliance with the requirements hereinafter provided.

SECTION 2. That a board to carry out the purposes of this act is hereby created, to be known as the Board of Dental Examiners, to consist of five reputable dentists resident of and for three years last before appointment actively engaged in the practice of dentistry in the District of Columbia, to be appointed by the Commissioners of said District for terms of five years and until their successors are appointed; *Provided*, That the first five appointments shall be made for terms of one, two, three, four, and five years, respectively. A majority of said board shall constitute a quorum. Vacancies occurring in said board shall be filled by appointment of eligible persons for unexpired terms.

SEC. 3. That it shall be the duty of the Board of Dental Examiners, first to organize by electing one of their number president and one secretary, to provide necessary books and blank forms, and publicly announce the requirements of this act, and the time, place, and means of complying with its provisions within thirty days from its passage; second, to promptly certify to the health officer for registration all who are engaged in the practice of dentistry in said District at the time of passage of this act who apply therefor; third, to test the fitness and pass upon the qualification of persons desiring to commence the practice of dentistry in said District after the passage of this act, and certify to the health officer for registration such as prove, under examination in theory and practice of dentistry, qualified in the judgment of the board to practise dentistry in said District; fourth, to report immediately information of any violation of this act, and, annually, the transactions of the board to the Commissioners of the District of Columbia; *Provided*, That all graduates of dental colleges which require a three years' course of study shall be entitled to certificates upon payment of the certification fee, and without examination as to their qualifications.

SEC. 4. That it shall be the duty of every person practising dentistry in said District at the time of the passage of this act to make application to said board, in form prescribed by said board, for certification, and present the certificates thus obtained for registration to the health officer within sixty days from the passage of this act. Every such person so registering may continue to practise without incurring the penalties of this act.

SEC. 5. That persons desiring to commence the practice of dentistry in said District after the passage of this act shall first obtain a certificate of qualification from the Board of Dental Examiners, granted under authority conferred upon said board by Section 3 of this act, and present the same to the health officer for registration.

SEC. 6. That it shall be the duty of the health officer to register all persons presenting certificates from said board in a book kept for this purpose, and indorse upon each certificate the fact and date of such registration.

SEC. 7. That certificates issued and indorsed under the provisions of this act shall be evidence of the right of the person to whom granted to practise under this act.

SEC. 8. That any one who shall practise or attempt to practise dentistry in the said District without having complied with the provisions of this act shall be deemed guilty of a misdemeanor, and, upon conviction thereof, shall be fined not less than fifty nor more than two hundred dollars, and in default of payment of such fine shall be imprisoned not less than thirty nor more than ninety days, said fines, when collected, to be paid into the Treasury of the United States, to the credit of the District of Columbia; *Provided*, That nothing in this act shall be construed to interfere with physicians in the discharge of their professional duties, nor with students pursuing a regular uninterrupted dental college course or in *bona fide* pupilage with a registered dentist.

SEC. 9. That to provide a fund to carry out and enforce the provisions of this act the Board of Dental Examiners may charge such fees, not exceeding one dollar for each certificate and ten dollars for each examination, as will from time to time, in the opinion of said board, approved by said commissioners, be necessary. From such fund all expenses shall be paid by the board ; *Provided*, That such expense shall in no case exceed the balance of receipts.

Approved, June 6, 1892.

---

### ALUMNI ASSOCIATION, NEW YORK COLLEGE OF DENTISTRY.

The following were elected officers for 1892: President, J. Howard Reed, D.D.S., M.D.S., 1881, 32 West Nineteenth St., City of New York; First Vice-President, M. Chas. Gottschaldt, M.D., D.D.S., 1882; Second Vice-President, Alfred R. Starr, M.D., D.D.S., 1880; Secretary, Vincent M. Munier, D.D.S., 1888, 102 West Ninety-fifth St., City of New York; Treasurer, Zachary T. Sailer, D.D.S., 1880, 40 West Thirty-third St., City of New York.

*Executive Committee.*—Sherman B. Price, D.D.S., *Chairman*, 1880, 13 West Thirty-second St., City of New York ; H. J. Parker, D.D.S., 1883, and E. S. Robinson, D.D.S., 1889.

*College Committee.*—George A. Hull, D.D.S., *Chairman*, 1888; Arthur L. Swift, D.D.S., 1885; Wm. B. Davenport, D.D.S., 1888; S. E. Hamilton, D.D.S., 1887, and Louis C. LeRoy, D.D.S., 1887.

Vincent M. Munier,
*Secretary.*

---

### CONNECTICUT VALLEY DENTAL SOCIETY.

At the annual meeting of the Connecticut Valley Dental Society, held at Greenfield, Mass., June 1, 2, and 3, 1892, the following preamble and resolutions were unanimously adopted :

Whereas, Advertisements, cards, and notices by dentists referring to "teeth without plates," crown- and bridge-work, etc., frequently appear in the public prints ; and

Whereas, Such advertisements, cards, and notices are misleading to the public, in that they claim or imply that these devices are new, and that in constructing these appliances they possess a superior skill over other practitioners ; and

Whereas, These devices are not new, but have been constructed and applied for many years past by various members of the dental profession ; and

Whereas, The Code of Ethics governing Dental Societies says, "It is unprofessional to resort to public advertisements, cards, hand-bills, posters, or signs, calling attention to peculiar styles of work, lowness of prices, special modes of operating; or to claim superiority over neighboring practitioners," and that "dentists are frequent witnesses, and, at the same time, the best judges of the impositions perpetrated by quacks; and it is their duty to enlighten and warn the public in regard to them;" and

WHEREAS, The objects of Dental Societies are to cultivate the science and art of dentistry and all its collateral branches, to elevate and sustain the professional character of dentistry, and to promote among them mutual improvement; therefore

*Resolved*, For the information and protection of the public, this Society condemn such advertisements, cards, and notices as not only unprofessional, but usually deceptive either by statement or implication.

GEO. A. MAXFIELD, D.D.S.,
*Secretary.*

---

### THE MISSOURI DENTAL COLLEGE ALUMNI ASSOCIATION.

THE Missouri Dental College Alumni Association, Dental Department of the Washington University, St. Louis, Mo, gave their annual dinner, Wednesday evening, March 9, 1892, at eight o'clock, at Faust's. After partaking of the good things provided, President Walter M. Bartlett made the opening address. The following gentlemen responded to toasts:

Chancellor W. S. Chaplin; Dr. Henry H. Mudd, Dean; Dr. F. H. Achelpohl, Dr. DeCourcey Lindsley, Dr. J. C. Goodrich, and Dr. H. H. Reith. Remarks were also made by Drs. Conrad, Freidman, Prothero, Stevens, Fuller, and Harper.

The Association held its annual meeting at the College Building, commencement day, Thursday afternoon, March 10, at four o'clock, President Walter M. Bartlett in the chair. Eighteen names were proposed for membership and elected. The Association proceeded to elect officers for the ensuing year, which resulted as follows:

Dr. DeCourcey Lindsley, President; Dr. James B. Newby, Vice-President; Dr. G. W. Applegate, Secretary; Dr. Carl E. Schumacher, Treasurer.

*Executive Committee.*—Dr. W. M. Bartlett, Dr. J. B. Newby, Dr. P. F. Helmuth.

Meeting adjourned to meet next commencement-day.

DECOURCEY LINDSLEY,
*President.*

### LAKE ERIE DENTAL ASSOCIATION.

At the Twenty-ninth Annual Meeting of the Lake Erie Dental Association the following resolution was unanimously adopted:

*Resolved*, That the manufacturers of porcelain teeth be requested to reduce the price in the same ratio to the decline in that of platinum. The advance in the price of this metal having been the alleged reason for the increase in the price of teeth.

C. D. Elliott,
*Secretary.*

---

### DENTAL SOCIETY OF THE STATE OF NEW YORK.

At the Twenty-fourth Annual Meeting of the Dental Society of the State of New York, held at Albany on the 11th and 12th inst., the following officers were elected for the ensuing year:

President, W. W. Walker, New York; Vice-President, F. T. Van Woert, Brooklyn; Secretary, C. S. Butler, Buffalo; Treasurer, H. G. Mirick, Brooklyn; Correspondent, R. Ottolengui, New York.

Chas. S. Butler,
*Secretary.*

May 18, 1892.

---

### NORTHERN OHIO DENTAL ASSOCIATION.

The next meeting of the Northern Ohio Dental Association will be held in Akron, Ohio, the first Tuesday in May 1893.

The newly-elected officers are, President, W. H. Whitslar, Cleveland; Vice-President, S. B. Dewey, Cleveland; Corresponding Secretary, H. Barnes, Cleveland; Recording Secretary, L. P. Bethel, Kent; Treasurer, Chas. Buffett, Cleveland.

H. Barnes,
*Corresponding Secretary.*

---

### RECENT PATENTS.

A list of recent patents, reported specially for the International Dental Journal.

476,088.—Dental Flask. William E. Stiles, Philadelphia Pa., assignor of one-half to Mahlon Moyer, same place. Filed November 18, 1891.

476,125.—Dental Flask-Holder. Emory A. Bryant, Aspen, Col. Filed January 10, 1891. Renewed April 14, 1892.

476,496.—Hand-Piece for Dental Engines. Wm. M. Speakman, Philadelphia, Pa. Filed February 23, 1892.

476,605.—Artificial Tooth. Robert Brewster, New Barnet, England. Filed September 23, 1891. Patented in England May 1, 1890.

476,944.—Dental Engine. Rudolph M. Hunter, Philadelphia, Pa. Filed February 12, 1892.

477,076.—Dental-Engine Attachment. Henry E. Spencer, Spencer, Miss. Filed October 10, 1891.

477,225—Dental Drill. Carl Rauhe, Dusseldorf, Germany. Filed March 24, 1892.

477,411.—Dental Plugger. Andrew J. Harris, Chicago, Ill. Original application filed May 5, 1891. Divided and this application filed July 16, 1891.

477,619—Dental Plugger. Martin L. Bosworth, Warren, R. I. Filed February 11, 1890.

Trade-Mark. 21,230. Local Anæsthetic for Painless Extraction of Teeth. Howe & Dills, Carlisle, Ky. Filed May 4, 1892. Essential feature, the words "Surmodont Analgia."

---

### NATIONAL ASSOCIATION OF DENTAL EXAMINERS.

The annual meeting of the National Association of Dental Examiners will be held at Niagara Falls, Monday, August 1, 1892, at ten (10) A. M. All State Boards are invited.

Fred. A. Levy,
*Secretary.*

